# A Software Suite for Limb Volume Analysis Applicable in Clinical Settings: Upper Limb Quantification

**DOI:** 10.3389/fbioe.2022.863689

**Published:** 2022-05-30

**Authors:** Mauro Nascimben, Lorenzo Lippi, Nicola Fusco, Marco Invernizzi, Lia Rimondini

**Affiliations:** ^1^ Department of Health Sciences, Center for Translational Research on Autoimmune and Allergic Diseases-CAAD, Università Del Piemonte Orientale (UPO), Novara, Italy; ^2^ Enginsoft SpA, Padova, Italy; ^3^ Physical and Rehabilitative Medicine Unit, Department of Health Sciences, Università Del Piemonte Orientale (UPO), Novara, Italy; ^4^ Department of Oncology and Hemato-Oncology, University of Milan, Milan, Italy; ^5^ Division of Pathology, European Institute of Oncology IRCCS, Milan, Italy; ^6^ Dipartimento Attività Integrate Ricerca e Innovazione (DAIRI), Translational Medicine, Azienda Ospedaliera SS Antonio e Biagio e Cesare Arrigo, Alessandria, Italy

**Keywords:** 3D laser scanner, rehabilitation, freeware, upper limb anatomical shape estimation, 3D volume measurement

## Abstract

In medicine, tridimensional scanning devices produce digital surfaces that replicate the bodies of patients, facilitating anthropometric measurement and limb volume quantification in pathological conditions. Free programs that address this task are not commonly found, with doctors mainly relying on proprietary software. This aspect brings reduced reproducibility of studies and evaluation of alternative measures. A software package made up of three programs has been developed and released together with supporting materials to enhance reproducibility and comparisons between medical centers. In this article, the functions of the programs and steps for volume assessment were introduced together with a pilot study for upper limb volume quantification. This initial experiment aimed to also verify the performance of digital volumes derived from the convex-hull gift-wrapping algorithm and the alternative analysis methods enclosed in the software. Few of these digital volumes are parameter-dependent, requiring a value selection. The experiment was conducted on a small mixed-gender group of young adults without correction for factors like arm dominance or specific physical training. In the sample under investigation, the analysis confirmed the substantial agreement between the clinical and current configurations of digital volumes produced by the package (*R* ^2^ interval from 0.93 to 0.97, *r* ranged from 0.965 to 0.984); in addition, as a general consideration, gender appears as a variable that could influence upper limb volume quantification if a single model is built.

## 1 Introduction

Laser scanning (3DLS) is a well-established methodology to quantify the volume of anatomical segments of the human body (Redaelli et al., 2018; Skinner et al., 2021). It has reduced cost, and its portable equipment can be easily integrated into health professionals’ routine examinations for diagnosis and patient follow-up (Anghel et al., 2016; Oranges et al., 2019). For example, the limb measurement of both circumference and volume could be inferred from laser-scanned legs in patients with filarial lymphedema (Yahathugoda et al., 2017) or scanned arms in patients who suffer from lymphedema after breast-cancer-related therapies (Invernizzi et al., 2018). In both medical conditions, the localized accumulation of liquids in the limbs due to disruption in lymphatic drainage leads to an abnormal compression of the inner anatomical structures. This pathological status requires swelling quantification for the early diagnosis of lymphedema and its careful monitoring (Cheifetz and Haley, 2010; O’Toole et al., 2013). Other clinical applications of anatomical measurements from digital body reconstructions include leg ulcer monitoring (Marjanovic et al., 1998), obesity evaluation (Cau et al., 2016a), residual limb volume appraisal for prosthetics in amputees (Tantua et al., 2014; Ryniewicz et al., 2017; Armitage et al., 2019), or patient-specific orthosis (Thometz and Liu, 2019). Modes for human body volume estimation using non-digital techniques are time-consuming. An accurate volume calculation of the limbs involves the Archimedes principle based on the observation of water displacement (WD) after the patients’ limb immersion (Auvert and Vayssairat, 2002). At present, several authors have proposed WD as the clinical gold-standard measurement for limb volume assessment (Sander et al., 2002; Karges et al., 2003; McKinnon et al., 2007; Rabe et al., 2010; Yang et al., 2018); however, it could be impractical to handle in clinics or hospital environments despite its rigor in volume determination. The alternative is to subdivide the limb length into sub-sections and measure limb perimeter at each section. Therefore, the total volume is approximated using the geometric formula of the cone, summing results of each limb segment (circumferential method, CM) Kosir et al. (2001). This method, also called the partial frustum model, is commonly applied in clinics (Tánori-Tapia et al., 2020) but sub-optimal when related to WD (Deltombe et al., 2007; Gjorup et al., 2010). Moreover, it is operator-dependent and prone to protocol changes between different hospitals (Tierney et al., 1996; Ng and Munnoch, 2010). In particular, the tension applied while performing the circumferential measurement on patients’ limbs and the precise location of anatomical landmarks are critical aspects of this procedure, especially in edematous areas (Tidhar et al., 2015). Tape tension may alter the measured circumference up to 3% (Cheah et al., 1989), with doubts on the appraisal persisting even when a particular type of tape with a controlled tension spring was tested (Labs et al., 2000). In this scenario, augmented reality tools proved reasonable medical practice support to facilitate anatomic assessment for clinical decision-making, with early studies dating back to 20 years ago (Mayrovitz et al., 2000). These tools proved to be safe, highly sensitive, reproducible, and easy-to-use in evaluating the upper limb volume in different clinical settings (Man et al., 2004; Ridner et al., 2007; De Sire et al., 2020; Invernizzi et al., 2020). In addition, different acquisition techniques provided consistent results: limb volume calculated by 3DLS showed values compatible with the Perometer technique, a different 3D analysis technology that uses parallel-coupled light emitters and receivers to capture limb shape (Binkley et al., 2020). Besides, health professionals could infer limb volumes by reworking pre-recorded full-body digital surface maps from three-dimensional surface imaging like magnetic resonance imaging or computed tomography. However, a crucial limitation to the broad diffusion of these methods for a clinical anthropometric evaluation could be the lack of specialized free software tools for body measurement assessment.

In light of these considerations, this article aims to provide three freely downloadable computer programs to fill the aforementioned technological gap and compare different digital volume approximation approaches with anatomical data. The software kit consists of three apps with a minimalist design to promote their usage by all hospital operators, even those with low computer skills, thus simplifying the user learning curve. They can work as stand-alone programs or as more sophisticated analysis workflow components. The goal of the freeware is to facilitate the measurement of the physical properties of the human body (mainly the volume) from 3D maps by offering a simple software environment for data handling and digital surface elaboration.

## 2 Materials and Methods

This section has been subdivided into three branches: presentation of the software package and workflows, limb volume calculation from the digital perspective and the related outputs produced by the software, and description of the pilot study for upper limb volume quantification.

### 2.1 Software Suite Overview

The software kit was coded in Python 3.8 programming language, partially adopting Open3D (Zhou et al., 2018) and Trimesh (Dawson-Haggerty, 2019) libraries, and included three Windows executable apps named Edit 3D, Slice 3D, and Cut 3D (Nascimben, 2022). The package had been tested on Windows 10 systems:

1. Edit 3D
• Operations performed: mesh file import, limb selection, mesh manipulation, outlier point removal, mesh reconstruction after outlier removal
• Output: post-processed volume visualization, saves cropped arm geometry file (as mesh) and volume data report
2. Slice 3D
• Operations performed: cropped arm import, limb subdivision in sections orthogonal to a major axis, and visualization
• Output: saves a report with additional volumes calculated with geometric methods, limb skin surface, limb length, the distance between the proximal and distal slices, area and perimeter of each limb section
3. Cut 3D
• Operations performed: limb import as mesh or point cloud data (PCD), limb point cloud resampling from mesh, PCD artifact removal
• Output: volume of the limb, perimeter and area of equispaced limb segments along all three axes, saves the cropped or edited limb (as PCD) and volume calculation result

The software package offers hardware-independent data management because it can analyze several pre-recorded file formats coming from different sources.

#### 2.1.1 Apps Interface

**FIGURE 1:**
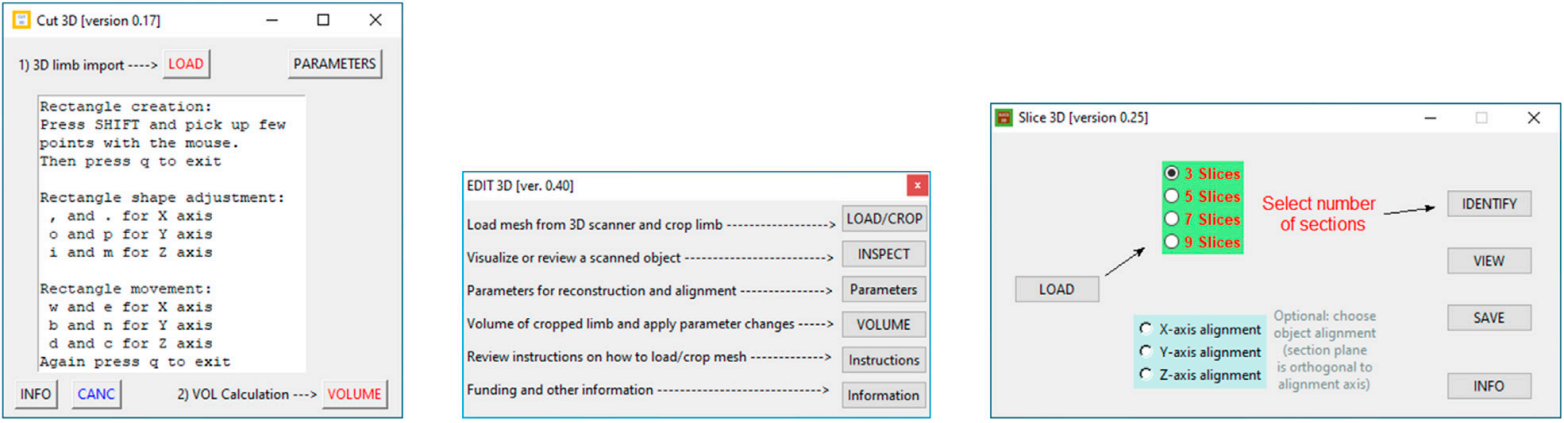
Cut 3D main window (left), Edit 3D interface (center), and Slice 3D appearance (right).

Graphical user interfaces of each program are portrayed in Figure 1. Edit 3D can import three mesh file formats: stereolithography (file extension is “STL”), object files (“OBJ” file extension), and polygon file format (“PLY” file extension, also known as Stanford polygon). Cut 3D can open the same file types as Edit 3D but also polygon file format files containing point clouds. Both Edit 3D (“Parameters” window) and Cut 3D (*Parameters* and *CANC* buttons) offer functions that can manage special operations on the input data. In addition, they can be combined into customized analysis sequences.

• Edit 3D main interface presents five buttons:
1. *Load/Crop* opens the scene file with texture and allows the user to crop the arm from the rendered scene. Thanks to texture visualization, the user can select points marked on the patient’s arm for a proper limb portion selection, discarding the background. The selected body part is saved on the hard drive as polygon file format (also known as Stanford triangle format) and used for all subsequent operations.
2. Users can review the cropped and saved limb by pressing the *Inspect* button. It is advantageous to identify possible outlier points; in fact, laser scanners might produce noisy scans in the presence of reflective surfaces or due to sensor errors.
3. Button *Parameters* provides a wide range of manipulation options on the limb geometry. In particular, when the user checks *Force resampling*, the program takes sampling and reconstruction quality values to control the sampling of points from the mesh with refinement by the Poisson disk sample sets (Yuksel, 2015). The resulting point cloud configuration benefits from distributing points equally in space, including advisable statistical properties. In addition, points could be automatically marked as outliers using distance from the neighbors (statistical method) or counting the number of neighbors in a sphere around them (few neighbors mean that point is an outlier). After outlier removal, the mesh could be rebuilt using the ball-pivoting (Bernardini et al., 1999) or screened Poisson reconstruction (Kazhdan and Hoppe, 2013) algorithms. Finally, the 3D object could be aligned to axis X, Y, or Z (coronal, transverse, and sagittal in medical imaging).
4. *Volume* button applies the manipulations selected in “Parameters,” shows the approximated volume of the post-processed mesh on display, and, if requested, automatically stores values in a text report
5. Buttons *Instructions* provide a quick guide of the commands, while *Information* reports the funding institution.
• The Slice 3D app main window contains commands to:
1. Import the cropped body segment as mesh data type (button *Load*).
2. Select the number of slices and the possibility of choosing the direction of the section planes (orthogonal to an alignment axis; the limb could be aligned along a major axis in Edit 3D).
3. A button called *Identify* executes background operations and shows the placement of the slices over the 3D body segment.
4. Button *View* allows the user to review sections in 3D and 2D. Each section is marked with a unique label to facilitate recognition.
5. Button *Save* stores a text file containing all calculations (additional volumes, skin surface, each limb section area, and perimeter). The report also includes the patient’s name and surname and a field accessible to the user for inserting notes.
6. Button *Info* contains information about the funding institution.
• Program Cut 3D was mainly conceived for point cloud data handling, its functionalities are as follows:
1. *Load* imports a mesh or a PCD data file: if mesh, it can display its texture.
2. The *Parameters* button allows the user to decide the adjustments of the bounding box during limb portion selection. Resampling values can increase points’ density and regulate the resolution of data while uniformly sampling from a mesh.
3. Button *CANC* allows the user to pick up artifactual points for elimination, automatically saving the new object.
4. Button *Volume* calculates the volume computing the minimal CH of the point cloud and saves volume as a text file and cropped limb as point cloud data file (polygon file format, with file extension “PLY”). In addition, it computes the volume with an experimental procedure explained in Section 2.2.
5. *Info* shows funding institution information.

#### 2.1.2 Interaction Between Programs

**FIGURE 2:**
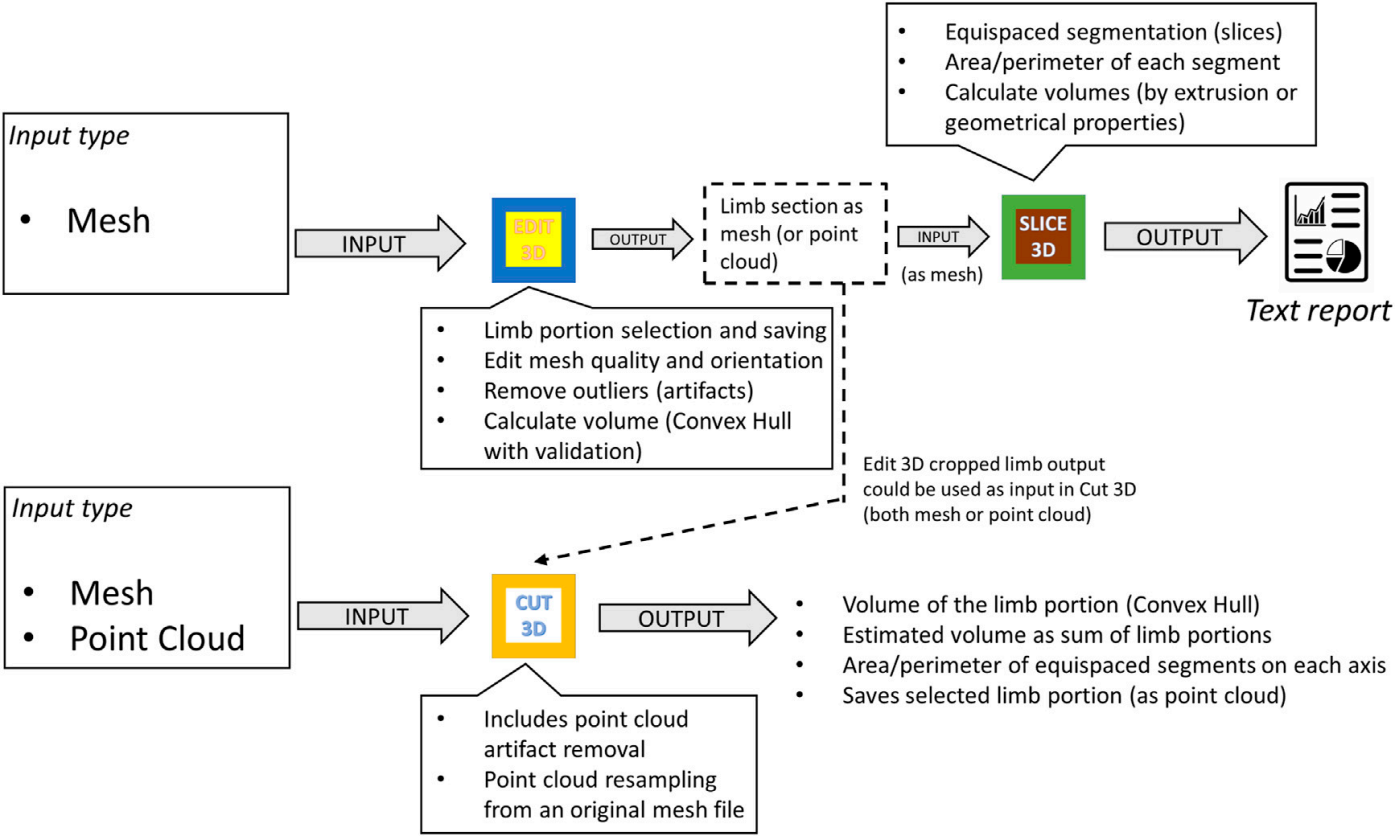
Workflow and interaction between software components of the kit.

Figure 2 exemplifies possible software kit workflows, showing apps inputs, outputs, and primary features. Apps were conceived to dialog between them or other software by sharing outputs: while Slice 3D works on meshes as input types only, Cut 3D and Edit 3D can switch to point cloud data. In this way, the software package offers tools capable of working with recording systems producing both meshes and point cloud data. Regarding the tasks performed, Cut 3D could be used as a “fast processing” app (lower part of Figure 2), whereas the combination of Edit 3D in sequence with Slice 3D allows the user to perform sequences of operations over the recorded limb; it could be interpreted as a “fine-tuning” pipeline for more complex analysis (upper part of Figure 2). All programs produce two types of files: text reports or edited geometries (in Stanford polygon). The output of Edit 3D could be a mesh or a PCD obtained resampling the input mesh. This operation could be helpful to convert a low-resolution object into a high-density PCD, later importable in Cut 3D. Another difference between Edit 3D and Cut 3D is how the user can select a limb portion for further analysis. While Edit 3D uses a computer mouse to draw the 2D window around the limb portion to be separated from the background scene, Cut 3D builds an initial 3D bounding box that the user can modify as a limb portion selector.

### 2.2 Digital Volume Assessment and Software Outputs

**FIGURE 3:**
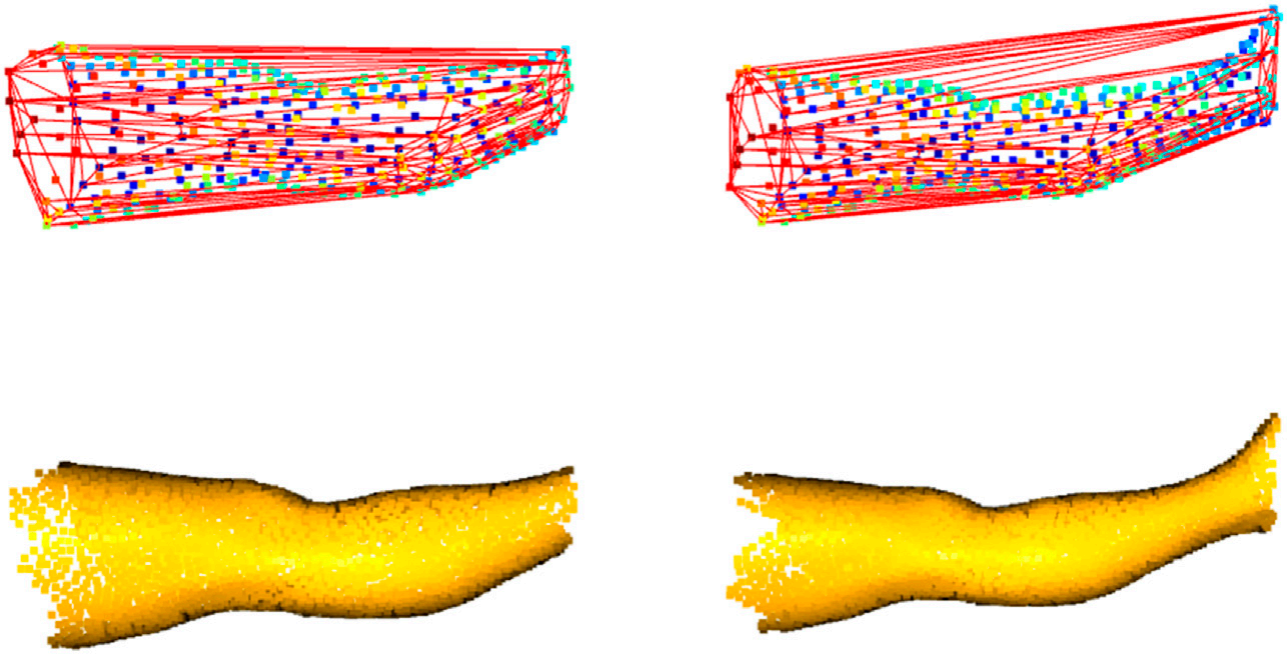
Convex-hull of two leg portions: shape enclosing the boundary points shows distortions, mainly when the limb includes the ankle (on the right).

Each program of the package computes the limb volume with a standard method derived from the CH estimation (gift-wrapping algorithm). The CH could be defined as the smallest volume polyhedral that contains a set of 3D points. Edit 3D and Slice 3D build the CH using the mesh elements (faces, edges, and vertices), while Cut 3D wraps the CH around the limb represented as a cloud of points. In addition, each program performs other measures or internal validation procedures. The CH-wrapping shape may overestimate limb volume, as shown empirically in Figure 3, and for this reason, further calculations based on geometry (non-CH digital volumes) were introduced in the software. Slice 3D generates a volume that applies the partial frustum model and two additional volumes based on the extrusion of limb circumferences. Extrusion of limb contours could be interpreted as a digital version of the disc model used for clinical limb volume evaluation (Man et al., 2004). In addition, one of the apps contains an internal validation routine that compares the post-processed CH volume with other volumes obtained by different digital mechanisms. This operation has been included to verify the consistency of the outcomes when the user carries out data manipulations.

Each program handles the calculations in a different manner:

1. In Edit 3D, the internal validation procedure of the CH post-processed volume (obtained after applying user parameter transformations by pressing the *Volume* button) compares it with the volumes built by the following custom functions:
• Function computing the Delaunay triangulation of mesh vertices or PCD, producing the total volume by summing the volume of all tetrahedrons that fill the digital surface inner space.
• Function that sums volumes of all tetrahedrons that fill the CH of the mesh or point cloud. It forces the construction of a CH searching all points while building the initial simplex.
• A function that employs the mesh vertices or PCD trying to surround the neighbors with an alpha shape (Edelsbrunner et al., 1983). Alpha shapes were built with 10 evenly spaced alpha parameters ranging from 0.35 to 0.05 (interval determined empirically) and volume returned as mean or median of values collected excluding outliers (exclusion by the interquartile range of 25–75% for the median or by z-scoring volumes and excluding values over two standard deviations). The exclusion of volume outliers was implemented to ignore volumes obtained by alpha shapes with cavities.
• A custom function that applies the incremental CH algorithm. It adds points for CH generation one by one. If the incremental CH algorithm encounters non-manifold edges, an issue is raised on the text report and volume replaced by mesh’s CH. When this happens, the user could select “Force resampling” to induce the creation of a new point cloud sampled from the original mesh and repeat calculations on this object type. Usually, this workaround solves the problem.

Internal validation is passed if the post-processed volume and the mean of all others are the same value up to the sixth decimal. All volumes and validation results are printed on the text file created by Edit 3D for the user’s final check.

2. Slice 3D *Save* button generates a text report containing the following information:
• Patient name, surname, and notes (this text field could include recording date, patient birthdate, or other details).
• The standard volume computed building the minimal CH of the limb portion.
• Volume obtained by the frustum formula using a certain number of equispaced slices (number of slices fixed to 100) orthogonal to the alignment axis as bases of the geometry and summing up the frustums’ volumes.
• Volumes obtained by the half-way extrusion of nine slices (on both directions) or by running extrusion over 100 slices generated along the limb length (starting from the lower limb extreme point).
• Skin surface area is computed by summing the mesh face area or by taking mesh boundaries as trapezoid sides, with limb extremities as the parallel edges of the trapezoid, and computing the trapezoid area.
• The number of slices inserted in the text report for area and perimeter calculation is decided by the user in the Slice 3D interface. Also, the direction of the cuts is user-dependent and is reported in the report.

In the actual version of Slice 3D, the software employs a pre-determined number of sections along the limb length for frustum and extrusion volumes (100 for both). This value was fixed to a high count for testing purposes in the pilot experiment but will be customizable in future versions. All non-CH volumes of Slice 3D rely on limb contours extracted from the limb mesh. If the program finds gaps in the slices’ contour, they are automatically filled iteratively by searching for the minimum segment closing the circumference. Whenever the program silently activates this procedure, it may require more time to complete it.

3. Cut 3D calculates the minimal CH of the point cloud (or mesh vertices) and includes an experimental method that sub-divides the limb into portions and sums up the CH volume of each portion.
• The experimental methodology attempted to optimize volume calculation in the presence of limb volume overestimation by the standard CH. It was created to compensate for the sinuosity of specific shapes like the knee or ankle. The number of sub-volumes will be adjustable in the next release of the software, but in this work, it is fixed to nine (app version 0.17), similarly to the number of subsections in the CM model.

Table 1 contains the digital volumes saved by the apps in their text reports. All standard CH volume methods use the QHull library in the back-end (Barber et al., 2013), while the other functions are custom scripts and four are parameter-dependent (Slice 3D non-CH volumes and the Cut 3D experimental one). In this initial version of the software package, parameters were kept similar to enhance performance comparisons (both CE CH and S 9 use nine limb portions, while S I and S F work on 100 partitions of the scanned object).

**TABLE 1:** Digital volumes created by the apps.

Program	Description	Abbrev
Cut 3D	Standard CH volume of PCD	C CH
Cut 3D	Volume as sum C.H.s from nine limb portions	CE CH
Slice 3D	Standard CH volume of mesh	S CH
Slice 3D	Volume from nine slice extrusions (half-way in both directions)	S 9
Slice 3D	Volume from 100 slice extrusions from lower extent upwards	S I
Slice 3D	Volume employing frustum formula with 100 slices as bases	S F
Edit 3D^a^	Sum of tetrahedrons from Delauney triangulation	
Edit 3D^a^	Alpha shape evaluations and volume selection	
Edit 3D^a^	Volume of CH with different initial simplexes	
Edit 3D^a^	Incremental CH algorithm	
Edit 3D	Standard CH volume applying user parameter transformations	E CH
Edit 3D	CH volume of initial object before processing	
		

a Part of the internal validation procedure.

### 2.3 Pilot Study: Upper Limb Volume Evaluation

The experimental procedure employed in this preliminary evaluation was already demonstrated in the previous literature (Cau et al., 2016b; Invernizzi et al., 2020). In both articles, the authors compared the arm volume derived from laser scansions to the anthropometric CM, including a cohort of normal subjects in their study. The selected CM approach required that an operator manually measured limb circumferences at equally spaced points along the arm starting from the wrist, and then the volume was obtained by the partial frustum formula discussed in Sitzia (1995). However, both studies make use of commercial systems. Similar hardware based on the 3DLS technology has been probed in the present work, but analysis relied on a zero-cost medium.

#### 2.3.1 Recording System

The hardware setup selected for running the experiment included a laser-scanner Structure Sensor Mark II (Occipital Inc., Boulder, CO, United States ) mounted over an iPad Air 2 64 Gb tablet (iOS 14.6 operating system). The depth resolution of the sensor was 1,280 × 960, calibrated by the manufacturer, and the operational range from 0.3 to 5 m using a sensor auto-exposure mode and gain set to 4 times. All scans were recorded by the freely available app distributed with the Structure Sensor, downloadable from the Apple App Store. The Structure Sensor app exports all morphological data to a mesh file in “OBJ” format accompanied by a separate texture file of the scanned arm. The original unit of measure of the 3DLS scene was in meters and later converted into cubic decimeters at volume reckoning.

#### 2.3.2 Data Collection

An experiment has been conducted on 17 healthy volunteers (9 males and 8 females, aged 24.41 ± 4.89 years, height 1.72 ± 0.10 m, weight 65.05 ± 14.02 Kg) to verify volumes produced by software kit programs. The 3DLS upper limb scans were recorded at the UPO Physical and Rehabilitative Medicine Unit (Alessandria, Italy). Upper limb data acquisition followed the instructions found in Invernizzi et al. (2020) for patient position and room environmental conditions. The recording device was in lateral view of the subject at a distance of 1 m. The subject was standing; the arm was positioned with the shoulder at 90 degrees of forward flexion and 0 degrees of horizontal abduction, with the elbow fully extended and palm oriented toward the floor. A trained physician applied the circumferential clinical method to calculate arm volume in all subjects. During CM assessment, nine segments had been marked with a dermographic pencil over the subjects’ upper limbs and recorded by the 3DLS to repeat digital volume calculations on the same arm portion evaluated by the clinical expert. Limb segments were measured starting from the pisiform bone using a ruler tape and fixing the inter-distance between segments at 5 cm. The medical doctor calculated the volume with the frustum formula, converting each segment’s limb circumference into the surface base of the cone, and summed the nine segments’ volumes to get the total volume of the arm portion. The CM-derived clinical volume constituted the ground truth (i.e., real-world information obtained on-site) to correlate with apps’ digital volumes. Apps calculations were managed by another person and independently from the clinical evaluation. The upper limb portion containing the CM markers was selected with Edit 3D app and later imported in Slice 3D and Cut 3D, repeating this operation for all subjects. Afterward, clinical results and volumes from the software were collected for statistical inference. Due to one corrupted file, the right arm of subject seven could not be processed correctly and was excluded from further analysis. Both left and right arm data were analyzed together without considering direct factors conditioning volumes like dominance, physical activity, or gender to verify the robustness of the digital techniques in the presence of a heterogeneous dataset. Applying internal software functions, all scanned arms were aligned toward the *X*-axis to ensure standard positioning of the 3D objects.

#### 2.3.3 Supplementary Parameter-Dependent Digital Volume

One additional custom output from Edit 3D, labeled “Resampled” (ER CH), has been added to represent the arm volume computed after resampling the original mesh. This supplementary digital volume was included to evaluate the impact of this transformation on the outcomes of the pilot study. Original mesh resampling to a PCD has been accomplished by using 7,000 uniformly sampled points and 250 points as the parameter for sample elimination (to reduce possible clusters created by uniform sampling). Both values were inserted in the Parameters windows of Edit 3D for automatic data processing by the software.

## 3 Results

This section documented the pilot study data analysis evaluating the behavior of digital volumes of the free software package compared to CM values extracted from upper limbs. Table 2 summarizes the descriptive statistics of digital volumes and their clinical counterpart, while Tables 3 and Table 4 present linear regression modeling and correlation. The Bland–Altman (BA) analysis, a classic method to determine the degree of agreement in clinical measurements, has been carried out and is shown in Figures 5, 6. Advanced statistics such as mediation analysis and common language effect size (CLES) tried to explain the findings furtherly for a profound data interpretation.

**TABLE 2:** Data summary (unit of measure in *dm* ^3^).

Program	Volume	Count	Mean	Median	St. Dev	Min	25%	50%	75%	Max
Cut 3D	C CH	33	2.09	1.94	0.54	1.34	1.64	1.94	2.68	2.94
Cut 3D	CE CH^a^	33	1.64	1.48	0.43	0.97	1.28	1.48	2.06	2.38
Slice 3D	S F^a^	33	1.66	1.50	0.43	1.03	1.34	1.50	2.11	2.48
Slice 3D	S CH	33	2.05	1.91	0.53	1.28	1.61	1.91	2.61	2.84
Slice 3D	S I^a^	33	1.63	1.50	0.42	0.99	1.33	1.50	2.08	2.46
Slice 3D	S 9^a^	33	1.65	1.52	0.43	1.01	1.33	1.52	2.10	2.47
Edit 3D	E CH	33	1.97	1.84	0.51	1.23	1.54	1.84	2.51	2.74
Edit 3D	ER CH^a^	33	1.93	1.80	0.50	1.21	1.52	1.80	2.45	2.70
	CM	33	1.85	1.63	0.49	1.23	1.46	1.63	2.35	2.70
										

a Parameter-dependent volumes.

**TABLE 3:** Linear regression models: digital volumes vs circumferential measurement.

Volume	Coeff	SE (*dm* ^3^)	*t*	*p* ≥∣*t*∣	*R* ^2^	Adj *R* ^2^	C.I. [2.5%]	C.I. [97.5%]
C CH	0.9	0.03	29.84	∗	0.97	0.97	0.84	0.97
CE CH	1.1	0.05	21.01	∗	0.93	0.93	0.99	1.21
S F	1.12	0.05	23.6	∗	0.95	0.95	1.02	1.21
S CH	0.92	0.03	30.81	∗	0.97	0.97	0.86	0.98
S I	1.12	0.05	20.84	∗	0.93	0.93	1.01	1.23
S 9	1.11	0.05	20.57	∗	0.93	0.93	1	1.22
E CH	0.95	0.03	30.24	∗	0.97	0.97	0.89	1.02
ER CH	0.97	0.03	30.78	∗	0.97	0.97	0.91	1.04
								

Coeff. is the regression coefficient, SE, mean standard error; C.I., mean confidence intervals.

**TABLE 4:** Correlation digital volumes vs circumferential measurement.

Volume	*r*	*r* ^2^	Homoscedasticity
C CH	0.98	0.97	Yes
CE CH	0.97	0.93	Yes
S F	0.97	0.95	Yes
S CH	0.98	0.97	Yes
S I	0.97	0.93	Yes
S 9	0.97	0.93	Yes
E CH	0.98	0.97	Yes
ER CH	0.98	0.97	Yes
			

### 3.1 Descriptive Statistics

As shown in Table 2 containing descriptive statistics, volumes were referenced with abbreviations to shorten their descriptions in the reports (using syntax introduced in Table 1). Neither the digital volumes nor CM values followed a normal distribution (Shapiro–Wilks test).

### 3.2 Clinical vs Digital Volume Modeling

The relationship between ground truth and digital volumes underwent linear regression analysis, with the findings collected in Table 3: the probability that the model coefficient was estimated by chance was marked by an asterisk if below 0.0001.

Table 4 contains the non-parametric equivalent of Pearson correlation, addressed by permutation testing and number of permutations set to 9999, with correlation by chance probability below the significance threshold for all digital volumes (magnitude 0.0001). Assumption of equal variances was tested by Levene statistics. In Table 4, the correlation coefficient *r* is reported together with its squared value *r* ^2^.

**FIGURE 4:**
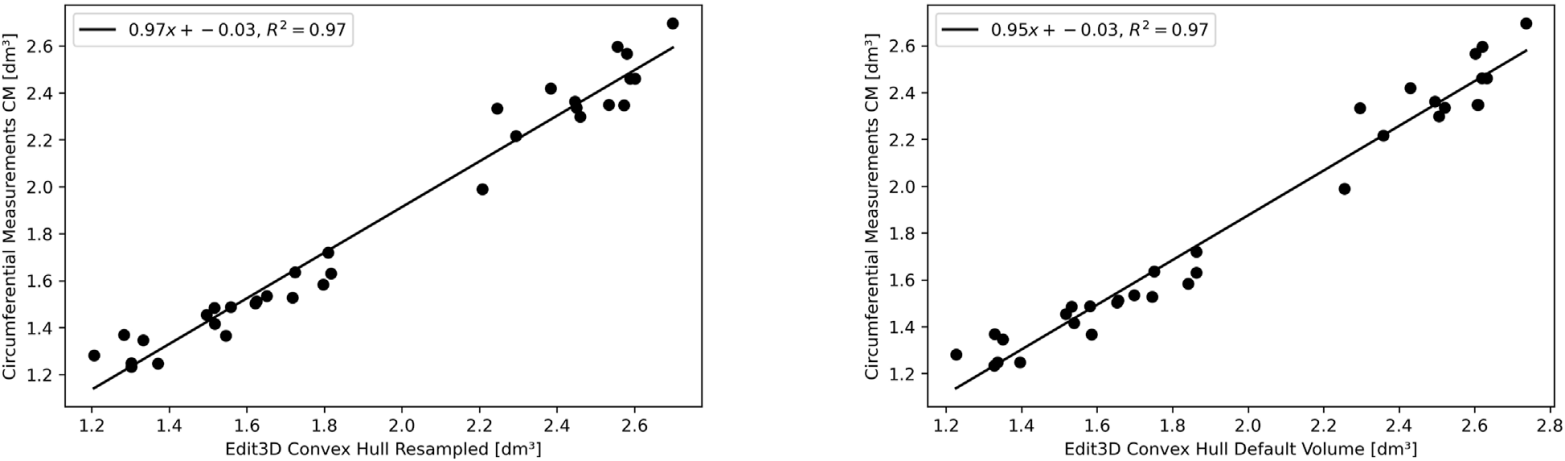
The linear relationship between clinical volume estimation by circumferential measurements CM with Edit 3D convex-hull volume (digital volume from mesh, on the right panel), and with Edit 3D resampled convex-hull (digital volume from point cloud data, on the left panel).

The experimental upper limb investigation with our 3DLS setup stated that standard volumes computed by tridimensional CH have a closer relationship to CM measures (coefficient of determination *R* ^2^ = 0.97). However, non-CH measurements with current parameters ranged from 0.93 to 0.95, exhibiting good performance matching CM values. Notably, the resampling operation does not affect volume outcomes compared to the CM ones. As an example, in the left image of Figure 4, the Edit 3D resampled volume produced by the PCD extracted from the initial limb mesh was regressed against CM volumes. On the right picture (Figure 4), the volumes were produced by the Edit 3D standard volume quantification by CH using the original upper limb mesh. A closing remark regarding the assumptions of linear regression: all residuals followed a normal distribution, checked by the Jarque–Bera test, and residual variances were homogeneous, as proved by the Breusch–Pagan test.

### 3.3 Bland–Altman Plots

**FIGURE 5:**
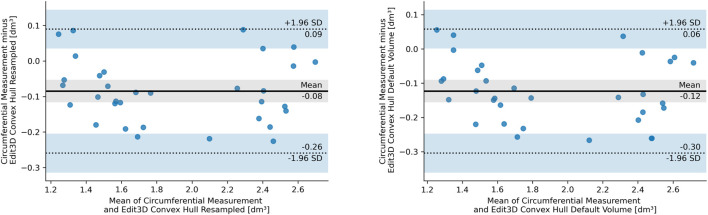
Bland–Altman plot of the differences between circumferential measurements and Edit 3D default convex-hull volume (E CH from mesh, right panel), and Edit 3D resampled convex-hull volume (ER CH from point cloud data, left panel). In the pictures, SD stands for standard deviation, dashed lines are the 95% limits of agreement, and blue bands indicate their confidence intervals. The gray-shaded areas are confidence intervals of the mean difference.

The BA analysis determines the degree of agreement between two clinical measurements based on their differences, usually depicted in the form of a graph (Giavarina, 2015). Figure 5 illustrates the BA analysis for the differences between two digital volumes and CM clinical measurements. A preliminary assumption of BA is that the differences should exhibit a normal distribution, which was the case of quantities in the plot. Indeed, all CH-derived volumes had differences between measurements that followed a normal distribution verified with the Shapiro–Wilks test. The figures show the equivalence plots of the digital volumes obtained from the mesh or a PCD, compared to CM, demonstrating the small impact (difference of 0.04 dm^3^) after data manipulation (from mesh to PCD). This consideration facilitates data transformation and interchange between the apps of the software kit in workflows that require specific treatment of the scanned objects (for example, artifact removal).

Furthermore, the Mann–Whitney *U* test confirmed that both ER CH and E CH follow the same distribution of values found in CM (ER CH vs CM U = 464.0, *p* = 0.304, and E CH vs CM U = 432.0, *p* = 0.150), with a slightly higher probability for ER CH under the null hypothesis. Indeed, BA shows a lower bias for ER CH than for E CH. The statistical outcome was not confirmed for the other digital volumes: a visual interpretation of this finding had been inserted as Section 3.3.1. The equality of variance assumption was checked by the Levene statistics, and it was valid for all comparisons between CM and digital volumes.

#### 3.3.1 Bland–Altman Limits of Agreement for the Difference Between Digital and CM Volumes

**FIGURE 6:**
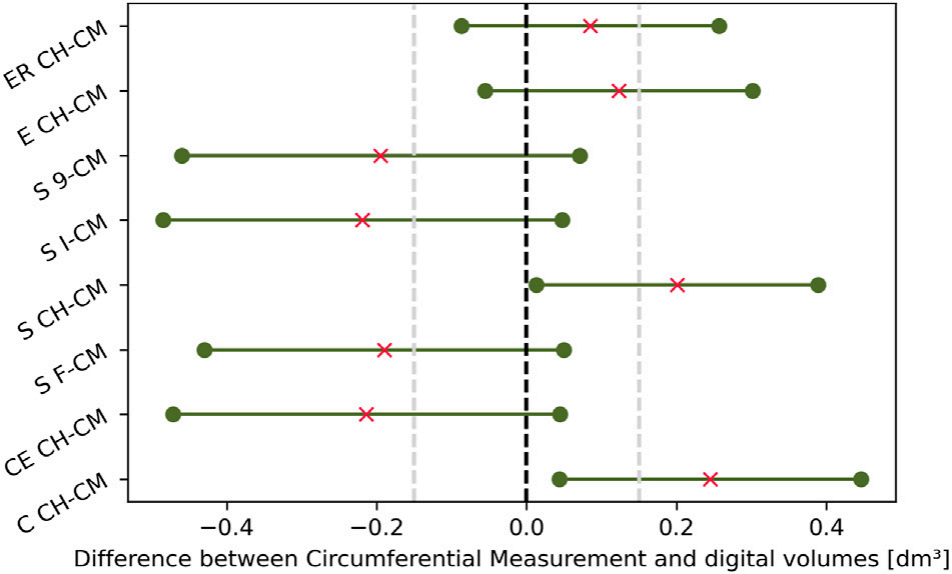
Bland–Altman limits of agreement for the differences between circumferential measurement CM and digital volumes, with the mean differences as red crosses. Differences between circumferential measurements and Slice 3D non-convex-hull volumes (S 9, S I, and S F) break the normality assumption of the Bland–Altman analysis.

The BA limits of agreement for the mean difference, calculated with the approximate method (Altman and Bland, 1983) and confidence intervals at 95%, have been plotted altogether as parallel lines for each digital volume and CM (Figure 6). However, the picture also includes the three digital volumes, which did not satisfy the normalcy requirement for the BA analysis; for this reason, the figure serves for illustrative purposes only, not allowing for a CH vs non-CH fair interpretation. The gray dashed lines are theoretical equivalence margins considering the CM technique’s minimal detectable change of 0.15 dm^3^ (converted from Taylor et al. (2006)). When two consecutive CM measurements on the same patients differ from that value, no change in volume can be assumed, and equality of the measurement could be hypothesized. Digital volumes whose mean difference with CM falls inside this region might be considered correspondent to CM upon adopting this empirical criterion. This proposition is demonstrated statistically because the Mann–Whitney *U* test for ER CH vs CM and E CH vs CM does not reject the null hypothesis that the two sets of measurements were drawn from the same distribution (as established in Section 3.3).

### 3.4 Mediation Analysis

**FIGURE 7:**
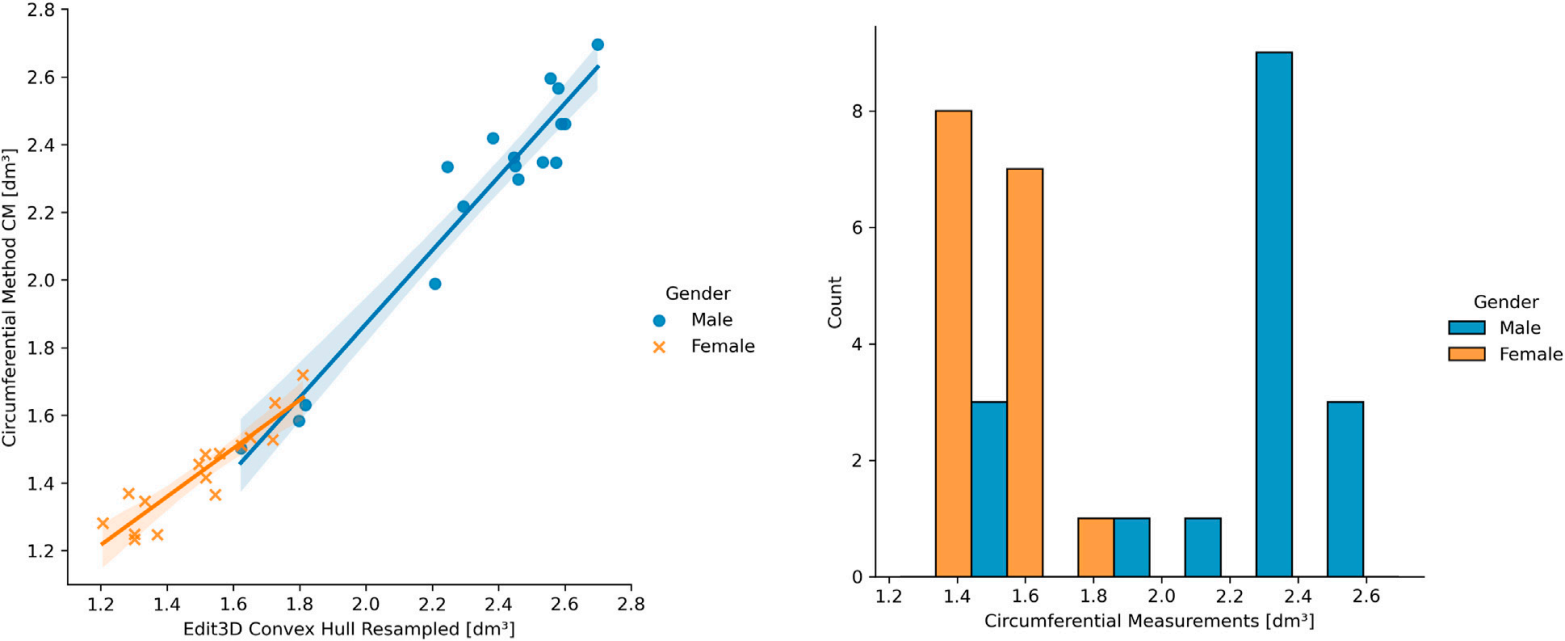
On the left: example of gender differences in the upper limb volume clinical vs digital. On the right: ground-truth data distribution among genders.

It was possible to visually identify two data clusters from the regression plots during linear modeling (Section 3.2), probably reflecting the arm shape differences between genders. The following pictures have investigated this aspect, subdividing ER CH data of Figure 6 by gender. In the left panel of Figure 7, the two groups display a slightly different behavior of the regression line. This confirms that distinct models might be more appropriate than a single linear or non-linear regression. Furthermore, the allocation of ground-truth values (i.e., the circumferential measurement) between males and females is shown in the right part of Figure 7. The effect of the gender variable in the relationship between clinical and digital volumes has been explored using mediation analysis, a statistical procedure borrowed from epidemiology. It adds a multilinear regression that includes gender (as mediation variable) into the clinical vs digital linear model to detect indirect associations (Table 5). The number of bootstrapping operations was set to 1,000. Intriguingly, all multilinear models derived from CH are sensible to the gender variable, while there is no indirect effect on others.

**TABLE 5:** Mediation analysis.

Volume	Direct Model				
	Estim. Coeff	Standard Error (*dm* ^3^)	Conf. Int. [2.5%]	Conf. Int. [97.5%]	*p* < 0.05
C CH	0.949	0.061	0.824	1.073	∗
CE CH	0.744	0.071	0.598	0.890	∗
S F	0.794	0.065	0.660	0.928	∗
S CH	0.942	0.058	0.822	1.061	∗
S I	0.778	0.073	0.629	0.928	∗
S 9	0.781	0.074	0.628	0.933	∗
E CH	0.902	0.057	0.785	1.019	∗
ER CH	0.896	0.056	0.781	1.010	∗

Volume	Indirect model				
	Estim. Coeff	Standard Error (*dm* ^3^)	Conf. Int. [2.5%]	Conf. Int. [97.5%]	*p* < 0.05
C CH	1.438	11.416	−0.688	6.817	∗
CE CH	1.273	11.227	0.057	9.293	∗
S F	0.640	5.047	−1.580	4.015	
S CH	1.326	10.928	−0.493	7.470	∗
S I	0.625	5.289	−1.653	4.688	
S 9	0.701	5.363	−1.406	4.254	
E CH	1.326	11.044	−0.016	7.555	∗
ER CH	1.206	9.758	−0.263	6.333	∗
					

### 3.5 Common Language Effect Size

In addition, the right-tailed Wilcoxon test with CLES has been conducted to understand the degree of overestimation given by digital volumes compared to CM ones (Table 6). In this pilot study involving a small group of young adults, there is a 34–35.4% probability that upper limb volumes not employing the CH will overestimate the CM volume. The Cut 3D “Estimated” CH volume (sum of sub-volumes) follows the same pattern, while the other CH volumes obtained from the whole object are more subject to overestimation (57.3–66.1% of the comparisons).

**TABLE 6:** Wilcoxon common language effect size.

	C CH	CE CH	S F	S CH	S I	S 9	E CH	ER CH
CLES	0.661	0.337	0.354	0.651	0.345	0.359	0.603	0.573
								

## 4 Discussion

The software suite proposes distinct analysis and editing techniques for scanned limb volume quantification. In the upper limb validation on healthy volunteers, it has been found that CH-based methods have the highest correlation with clinical volume assessment. However, the ground-truth methodology applied is a sub-optimal method because the limb circumference measured with a rule tape or caliper is subject to both inter-operator and intra-operator variations. Thus, it cannot be considered a top-notch estimation, even if it has positive aspects like simple implementation. On the opposite, the volume deducted by WD after limb immersion might be more precise but challenging to investigate during the clinical routine, considering the possible presence of cutaneous infections (Grada and Phillips, 2017). For example, in edematous patients after cancer treatment, WD and CM clinical methods for upper limb volume assessment, even if correlated, were partially discordant (Sander et al., 2002). The literature presents uncertainty on CM values that tend to be smaller than the most reliable WD measurement (Adriaenssens et al., 2013) or, in other works, larger than WD (Taylor et al., 2006). In this latter study, the authors also proposed the adoption of a standard margin of error for the CM methodology by accepting a volume change only if the measurement differs by 150 ml (equivalent to 0.15 dm^3^). The authors of Meijer et al. (2004) proposed to apply the CM method only for patient’s lymphedema follow-up and not as a WD replacement for diagnosis. In addition, the literature of CM procedures encloses CMs that apply different techniques: disc model, the frustum sign model, and the partial frustum model, and in the studies employing the latter, a different number of limb sub-divisions may be found. Laser scanners provide an alternative to real-world measurements, and their usefulness has already been proved inside hospitals. Most of them work under commercially licensed software for processing and analysis, limiting data exchange and reproducibility of the results between laboratories. Offering a free software kit could be an alternative to proprietary programs, allowing clinicians to have a wide range of possibilities while conducting analysis and limb volume estimation. In the volume validation experiment, upper limbs were preferred because they have a stable tubular shape between subjects. On the contrary, the volume of the lower limb holds higher variability and probably does not find a valid clinical counterpart in the CM method because circumference measurement and volume might not show a direct correlation (Guex and Perrin, 2000). In the control group of 14 healthy females in Cau et al. (2016a), a *r* ^2^ = 0.83 had been found between CM and 3DLS lower limb volumes. In Tan et al. (2013), the authors found that the Perometer’s infrared optoelectronic volumes tend to underestimate CM values of lower limbs. The experimental digital volume produced by Cut 3D had been conceived to accommodate the wavy silhouette of the legs at the joints, given the possibility of sub-dividing the shape into sub-CHs, and future experiments will corroborate this hypothesis. Also, the doubt that curvy morphology might be overestimated by digital CH methods persists on upper limbs. Wilcoxon CLES results demonstrated that non-CH volumes are less subject to overestimating CM values, extending the findings to other interpretations. Indeed, non-CH measures were introduced in the software to compensate for the possible overestimation of limb volume caused by the CH shape-wrapping algorithm (Figure 3). The CM being a sub-optimal measurement, it could be interesting to understand if clinical WD volumes find a valid digital counterpart in CH or non-wrapping volumes. For example, in Preuß et al. (2018), WD values appeared more prominent than digital ones in healthy arms of patients affected by cancer-related secondary lymphedema.

### 4.1 Comparison With Similar Works

Linear regression and correlation analysis between digital volumes and CM were compatible with upper limb experiments run on a comparable group of normal subjects using the same CM method and 3DLS volume from commercial software (Cau et al., 2016b; Invernizzi et al., 2020). Table 7 recaps these outcomes, but readers should deem the several factors influencing Pearson correlation results, for example, the different characteristics of the groups (Goodwin and Leech, 2006). Correlation alone cannot substantiate the interchangeability of two quantities but support their validity as they follow the same measurement trend of the underlying limb volume.

**TABLE 7:** Healthy subject upper limb volume: digital volumes vs circumferential measurement.

Ref	Software	Sample Size	Age	Gender	*r* ^2^	H^a^	Norm^b^
Cau et al. (2016b)	Comm	12	29 ± 5.39	–	0.923	–	Yes
Invernizzi et al. (2020)	Comm	30	27.6 ± 9.8	M = 14,F = 16	0.99	–	–
Current work	Free	17	24.41 ± 4.89	M = 9,F = 8	0.97^c^	Yes	No
							

a Homoscedasticity.

b Data follow a normal distribution.

c C CH, S CH, E CH, ER CH.

### 4.2 Notes on Linear Modeling

The different patterns at linear regression between genders (Figure 7, left panel) suggest that a single linear model cannot entirely describe a heterogeneous sample of young adults, and genders should be separated when building a clinical protocol for patient limb volume evaluation. The presence of two subpopulations with different characteristics may also explain the lack of gaussianity (Figure 7, right panel), as found during the normalcy analysis. Considering this observation, separate linear models for females and males could capture a higher correlation between 3DLS and CM volumes. During the discussion of the experimental results in Cau et al. (2016b), the authors argued the CM volume’s chance to slightly undervalue small arms and overrate large arms. Moreover, mediation analysis showed that a few digital volumes, those not derived from CH, suffer no indirect effect on the interrelations among CM, gender, and digital outcomes. Gender could be interpreted as involving a causal pathway between CM- and CH-based volumes. The CH gift-wrapping algorithm probably adapts to both gender shapes considering that women’s arms are thinner and closer to cylindrical shapes than men’s, where the muscular structure is dominant. This condition might be emphasized in young adults, and other factors like side dominance and specific upper limb sports training might play a role.

### 4.3 Further Restraints and Future Developments

The present work presented the software kit and volume outputs with an initial validation experiment. The pilot study has been carried out on a limited number of young adults, and it did not consider the influence of systematic errors on the measured values for both CM and digital volumes. It is known from the literature that operator-dependent variability affects CM results (Devoogdt et al., 2010), and this observation also holds for the digital volumes because the limb portion under analysis is selected manually. Future experiments will focus on enlarging the sample by stratifying the population for accurate volume validation and examining the patients’ clinical status from digital surface maps produced by 3DLS. Also, other anthropometric measures produced by the software, particularly skin surface and area of limb sections, will be related to specific pathological syndromes. The authors of Marmulla et al. (2003) and Gibelli et al. (2018) found that the surface area of the 3DLS facial anatomy is compatible with measurement from other techniques (stereophotogrammetry or pre-surgery optoelectronic surface registration). In addition, the development of this free software kit will encourage the testing of the programs by other researchers with different setups and clinical requirements. Given the broad range of outputs produced by the software, it could be advisable to validate the digital outcomes by selecting the appropriate app compatibly with the hardware available and optimizing the parameter-dependent digital volumes by finding the technique that better suits the recording conditions, input types, and clinical questions under investigation. For example, the digital volume configuration tested during the pilot study identified Edit 3D CH volumes as those likely to derive from the same distribution of values as CM and suitable for clinical application in similar studies. It should also be considered that several parameters have been fixed in this pilot study, for example, the number of slices for S F and S I, but the customization of these values and their optimization may lead to more accurate results for non-CH measures.

## 5 Conclusion

A free software kit for the morphological quantification of scanned limbs has been introduced, offering an alternative to commercial products for clinicians interested in analyzing anatomical shapes from digital scans. It could run limb volume appraisal, and in addition, the software can perform supplementary operations and measurements on recorded surfaces like outlier removal, morphology resampling, or slicing. Benefits of a free software package downloadable by health operators include an easy comparison of results between hospitals obtained by the same medium, enhancing experiment reproducibility, and data sharing between laboratories. In the context of the upper limb analysis of young adults, the pilot study showed that CH-based digital volumes have high reliability with the clinical CM method. It could have relevant implications in the clinical setting for health professionals involved in limb volume assessment in several pathological conditions. Therefore, future work will optimize digital volume parameters and address clinical studies.

## Data Availability

The present article contains all information to reproduce the results obtained during the upper limb volume quantification using the software suite. Supplementary materials, video tutorials, and user guides are available on the following website https://mn-visions.gitbook.io/software-kit-for-3dls-limb-volume-quantification/. In the online repository, readers can also find details regarding the software architecture and further information, including how to deal with the other functions of the programs. Each app of the software kit is downloadable as a zip file from Zenodo (10.5281/zenodo.5849851). The present analysis was carried out with the “launch-version” of the software. Future versions will be uploaded on the same platform.

## Abbreviations

3DLS, 3D laser scanning; WD, water displacement; CM, circumferential measurement; PCD, point cloud data; CH, convex-hull; BA, Bland–Altman; CLES, common language effect size; C CH, Cut 3D standard gift-wrapping volume from point cloud data; CE CH, Cut 3D volume as the sum of convex-hulls from limb sub-sections; S CH, Slice 3D standard convex-hull volume of the mesh; S 9, Slice 3D volume by bilateral slice extrusion; S I Slice 3D volume by upward extrusions; S F, Slice 3D frustum-type volume; E CH, Edit 3D standard volume; ER CH, Edit 3D volume after resampling (2.3.3).
